# Editorial: Progress and challenges in computational structure-based design and development of biologic drugs

**DOI:** 10.3389/fmolb.2024.1360267

**Published:** 2024-02-07

**Authors:** Traian Sulea, Sandeep Kumar, Daisuke Kuroda

**Affiliations:** ^1^ Human Health Therapeutics Research Centre, National Research Council Canada, Montreal, QC, Canada; ^2^ Computational Protein Design and Modeling, Computational Science, Moderna Therapeutics, Cambridge, MA, United States; ^3^ Research Center of Drug and Vaccine Development, National Institute of Infectious Diseases, Tokyo, Japan

**Keywords:** binding affinity, specificity, therapeutic antibody, developability, in silico method, molecular modeling, molecular simulations, machine learning

Biotherapeutics have emerged as a major class of pharmaceuticals, encompassing monoclonal antibodies, recombinant human proteins and enzymes, fusion proteins, antibody drug conjugates, multi-specific formats, peptides, and vaccines. These modalities serve a wide range of therapeutic areas, including immune-oncology, inflammation, cardiovascular, metabolic, infectious, and rare diseases (DeFrancesco, 2019; Kang and Jung, 2020; Lu et al., 2020; Kaplon et al., 2023). Recent advancements in structure determination, structure prediction, bioanalytical characterization, and machine learning have established *in silico* approaches as a key toolbox employed in the biologic drug discovery and development pipelines (Fischman and Ofran, 2018; Norman et al., 2020; Fernandez-Quintero et al., 2023). Additionally, physics-based molecular modeling and simulation methods, along with empirical linear models, have matured to routine implementation during biotherapeutic drug candidate selection and optimization. However, the accuracy of these predictions can be improved. Further refinements will be welcomed, particularly towards binding affinity predictions and developability assessments.

At the same time, with the fast-paced infusion of artificial intelligence in various research areas that impact daily life, we are witnessing a new chapter being written in biological drug design (Kim et al., 2023). A wide range of nonlinear models, from machine learning to unsupervised deep neural networks and language models, is now emerging. These models, fueled by still modest but expanding biological and structural datasets, are complementing classical methods (Baek et al., 2021; Jumper et al., 2021; Kryshtafovych et al., 2021; Bennett et al., 2023; Lin et al., 2023; Wodak et al., 2023). There are also increasing attempts to integrate advanced computational methods with next-generation sequencing, either from synthetic or natural libraries, to enhance the efficient hit-to-lead optimization and *de novo* discovery of tight binders with favorable developability profiles (Makowski et al., 2021; Mason et al., 2021; Hie et al., 2023; Parkinson et al., 2023). While past structure-based efforts were focused on optimizing existing antibody candidates, the emerging trend is the hopeful possibility of *de novo* discovery of antibody-based and other biologic drugs via *in silico* methods. This opens prospects for extensive application of computational techniques in biologic drug discovery and development.

This Research Topic comes at an opportune time when the accuracy limits are being pushed for classical methods and foundations are being established for machine learning methods. These complementary tools are poised to enhance the entire biologics drug development pipeline, from the molecular design and property optimization to large-scale manufacturability. In this Research Topic, readers will find a breadth of computational approaches, ranging from established 3D structure and physics-based techniques to innovative explorations in sequence-based non-linear machine learning models.

An overview of the current state and opportunities for synergistic use of computation and experimentation in this field is provided by Bauer et al. The authors described their vision of Biopharmaceutical Informatics and discuss already available computational methods at each stage of the biologic drug design-discovery-optimization-development pipeline. The authors have provided useful cues on how best to apply these *in silico* methods and how to combine them with experimental approaches to maximize the odds and efficiency of arriving at biologics that are both effective and developable.

Fundamental understandings of molecular properties of the drug candidates and their targets are essential to advance both biologic discovery and development. Di Rienzo et al. focused on discerning the rules that define antibody-antigen recognition as a fundamental step in the rational design and engineering of functional antibodies with desired properties. Their novel method, which is based on the 3D Zernike polynomials to generate shape and electrostatic descriptors capturing both global and local protein surface physicochemical properties, accurately classified types of antibody-antigen interfaces solely based on paratope surface information. Fernandez-Quintero et al. took a deep dive into seemingly similar interfaces between the various Ig-folded domains that make up a monoclonal antibody structure. Using classical MD simulations and analyses, they revealed and compared contact maps that can be used to inform selection of favorable point mutations for the design of bispecific antibodies. In their case study, Paul et al. described the well-recognized yet inadequately understood trade-off between binding affinity and thermal stability, which can have significant implications during the lead candidate optimization stage. Using classical force-field methods, molecular dynamics, and amino-acid hydropathy, they observed affinity-stability correlations and patterns in key pairs of residues called hotspots.

Novel tools are also reviewed in this Research Topic. For example, Jaszczyszyn et al. assembled a timely review of recent advances of deep-learning based tools for structural modeling the variable regions of antibodies. In addition to cataloguing underlying algorithms and benchmarking their performance, the authors offered their perspective on how the emerging high accuracy of antibody paratope modeling can influence the field of biologics drug discovery. Engelberger et al. provided the energy breakdown guided protein design (ENDURE) tool for accurately assessing energetic contributions from individual and combinatorial mutations to the overall protein stability. An interesting feature is the residue depth analysis which enables tracking the energetic contributions of mutations occurring in different spatial layers of the protein structure. Spoendlin et al. introduced the second iteration of their structural profiling of antibodies to cluster by epitope (SPACE) tool, which builds upon the recent progress in machine learning antibody structure prediction and a novel clustering protocol. It improved data coverage and identified even more diverse clusters of antibodies that bind to the same epitope. These tools are expected to further advance rational design of biotherapeutics.

Proof-of-concept studies illustrating novel screening campaigns that combine computational design with experimental data are also presented. Arras et al. combined next-generation sequencing of semi-immune/semi-synthetic libraries built on a humanized VHH framework with machine learning, data processing, and model building for simultaneous optimization of affinity and developability. The proposed typical early drug discovery methodology generated diverse and potent VHH hits against NKp46 protein without requiring further humanization and developability optimization, thereby accelerating drug discovery. Gaudreault et al. focused on protein-protein docking with flexible side chains while retaining rigid protein backbone to discover novel binders against predefined target epitopes. Their approach was applied to randomized libraries of surface mutations introduced in a rigid protein scaffold called DARPin, leading to the design and experimental validation of an enriched small set of hits against a predefined epitope on the BCL-W target protein.

The literature in this field is growing rapidly. Our Research Topic does not cover all computational aspects of biologic drug discovery. Nonetheless, the articles compiled here hopefully offer timely snapshots of key components along a biologic drug’s discovery, design, and development.
